# Third BNT162b2 Vaccine Booster Dose against SARS-CoV-2-Induced Antibody Response among Healthcare Workers

**DOI:** 10.3390/vaccines10101741

**Published:** 2022-10-18

**Authors:** Khetam Hussein, Halima Dabaja-Younis, Moran Szwarcwort-Cohen, Ronit Almog, Ronit Leiba, Avi Weissman, Michal Mekel, Gila Hyams, Nethanel A. Horowitz, Vardit Gepstein, Hagar Cohen Saban, Jalal Tarabeia, Michael Halberthal, Yael Shachor-Meyouhas

**Affiliations:** 1 Infection Control Unit, Rambam Health Care Campus, Haifa 3109601, Israel; 2 The Ruth & Bruce Rappaport Faculty of Medicine, Technion–Israel Institute of Technology, Haifa 3109601, Israel; 3 Rambam Health Care Campus, Haifa 3109601, Israel; 4 Pediatric Infectious Disease Unit, Ruth Rappaport Children’s Hospital, Rambam Health Care Campus, Haifa 3109601, Israel; 5 Virology Laboratory, Rambam Health Care Campus, Haifa 3109601, Israel; 6 Epidemiology Unit, Rambam Health Care Campus, Haifa 3109601, Israel; 7 Nursing Management, Rambam Health Care Campus, Haifa 3109601, Israel; 8 Department of Hematology and Bone Marrow Transplantation, Rambam Health Care Campus, Haifa 3109601, Israel; 9 Department of Pediatrics B, Ruth Rappaport Children’s Hospital, Rambam Health Care Campus, Haifa 3109601, Israel; 10 Nursing Faculty, The Max Stern Yezreel Valley College, Afula 30080, Israel

**Keywords:** antibody concentrations, booster, BNT162b2 vaccine, humoral response, SARS-CoV-2

## Abstract

This study assessed humoral response to the third BNT162b2 dose among healthcare workers (HCW). This prospective cohort study of HCW tested for anti-spike antibodies (LIAISON SARS-CoV-2 S1/S2 IgG assay) at 1, 3, 6, 9, and 12 months after receiving the second BNT162b2 vaccine dose (tests 1, 2, 3, 4, and 5, respectively). A third (booster) vaccination dose was introduced before test 4. Linear regression model was used to determine the humoral response following vaccine doses. For each serology test, changes in log-transformed antibody concentrations over time, adjusted for age, sex, underlying diseases, steroid treatment, and smoking were described using the general linear mix model. Serology tests were performed at 3, 6, 9, and 12 months after the second vaccine dose in 1113, 1058, 986, and 939 participants, respectively. The third dose was received by 964 participants before the 9-month tests, 797 of whom participated in the 9- and 12-month serology tests. A significant inverse correlation was noted between time from third dose and antibody concentrations (Spearman correlation −0.395; *p* < 0.001). Age (*p* < 0.0001; CI 95% −0.005–−0.004), heart disease (*p* < 0.0001; CI 95% −0.177–−0.052), immunodeficiency (*p* < 0.0001; CI 95% 0.251–−0.106), and smoking (*p* < 0.0001; CI 95% −0.122–−0.040) were significantly associated with decreased antibody concentrations. Female sex (*p* = 0.03; CI 95% 0.013–0.066) was associated with increased antibody concentrations. The third booster dose had a better effect on immunogenicity, with higher antibody concentrations among tested HCW. Heart disease, smoking, and other known risk factors were associated with decreased antibody concentrations.

## 1. Introduction

Since the initial outbreak of coronavirus disease 2019 (COVID-19) in December 2019, the pandemic has claimed more than 6 million lives [1]. With the availability of vaccinations, as of March 2022, 64% of the world’s population had been vaccinated worldwide [1].

Throughout Israel, the Pfizer-BioNTech (BNT162b2) COVID-19 vaccine was administered almost exclusively. Published studies from Israel related to a two-dose regimen of the BNT162b2 vaccine between December 2020 and April 2021 have reported on its efficacy, effectiveness, and immunogenicity [2,3].

A decline in immunity and efficacy was documented 6 months after introduction of the BNT162b2 vaccine [4,5]. As a result, many of fully vaccinated individuals became infected with severe acute respiratory syndrome coronavirus 2 (SARS-CoV-2) during the fourth wave of COVID-19 that began in July of 2021. Nevertheless, they had lower death and hospitalization rates as compared to unvaccinated people [5,6]. However, efficacy of the BNT162b2 vaccine against viral mutations was documented as lower and accompanied by waning immunity, which led to the decision to recommend a third BNT162b2 vaccine dose, to provide a “booster effect” [7].

By early August 2021 a third dose (“booster”) was approved in Israel for citizens over 60 years old and high risk patients. Soon thereafter, healthcare workers (HCW) and the rest of the population also were encouraged to receive a third dose. The effectiveness of the third dose on the Israeli population was rapid, with a marked decline in SARS-CoV-2 confirmed cases, and saw measurable protection against severe disease, hospitalization, and mortality compared to those who received only two doses of the BNT162b2 vaccine [8,9]. In addition, there were reports of improved efficacy and humoral immune response in high risk populations for the third BNT162b2 dose as compared to the two dose regimen [10,11,12].

Humoral response after the third BNT162b2 dose has been widely reported among high risk patients, particularly those after organ transplantation [13], bone marrow transplantation [14], or undergoing hemodialysis [15].

This study was aimed at assessing the humoral response to the third BNT162b2 dose among healthcare workers at a tertiary hospital in Haifa, Israel.

## 2. Materials and Methods

We conducted a prospective cohort study among fully vaccinated healthcare workers (HCW) and retired HCW at Rambam Healthcare Campus (RHCC), a 1000-bed university hospital serving the more than two million residents of Northern Israel. The only tertiary hospital in the region, Rambam Health Care Campus has 5520 employees: 1220 physicians, 1880 nurses, 1137 paramedical workers, and 1283 administrative workers.

All fully vaccinated HCW (two BNT162b2 vaccination doses) with no history of COVID-19 infection were invited to participate in the study. Those who consented underwent serial serological testing at 1, 3, 6, 9 and 12 months after receiving the second vaccination dose (during February, April, July, and October 2021, and January 2022).

Participants having a positive SARS-CoV-2 PCR test at any time during or before the study were excluded.

All participants completed a computerized questionnaire that included questions regarding demographic characteristics, comorbidities, medications, allergic reaction or rash following vaccination, confirmed COVID-19 infection, or flu-like illness at each testing time point.

During August 2021 a third vaccine dose (“booster”) was offered to all fully vaccinated individuals for whom 5 months had passed since their second dose. As a result, there were two distinct groups in the fourth and fifth testing time points at 9 and 12 months after the second dose: those who received three doses and those who received two doses.

The study was approved by the hospital’s Internal Review Board (#021-021), and written informed consent was obtained from all participants.

### 2.1. Serology Assays

Serology testing was performed at 1, 3, 6, 9 and 12 months post-vaccination on LIAISON^®^ XL analyzer with the LIAISON SARS-CoV-2 TrimericS IgG assay (DiaSorin S.p.A., Saluggia, Italy) according to the manufacturer’s instructions. This chemiluminescent immunoassay uses magnetic particles coated with recombinant trimeric SARS-CoV-2 spike protein for the quantitative determination of IgG antibodies. Cut-off values for positive serology were 22 AU/mL, border line 13–22 AU/mL; negative serology was reported for values < 13 AU/mL. When needed (values > 799 AU/mL), serum was diluted on-board 1:20 with LIAISON TrimericS IgG diluent.

### 2.2. Statistical Analysis

Descriptive statistics in terms of mean, standard deviation (SD), median, percentiles, and ranges were calculated for all study parameters. Pearson correlation was used to assess the differences between antibody concentrations and time from third dose. Linear regression model was used to describe the interaction (slope) between the time from third dose of the fourth and the fifth antibody concentrations compared to the interaction between time from the second dose and the first, second, and third antibody concentrations. The Log-transformed function was used since Kolmogorov Smirnov testing revealed an abnormal antibody concentration distribution. The general linear mix model with log-transformed antibody concentrations was used to describe the changes in antibody concentrations over time, with adjustments for age, gender, heart disease, active oncological disease, lung disease, systemic autoimmune disease, immunodeficiency, hypothyroidism, steroid, smoking, and first, second, third, fourth, and fifth antibody concentrations. The age of HCW served as a random effect while all other parameters were used as fixed effect; *p* < 0.05 was considered significant. SPSS version 27 was used for all statistical analyses.

## 3. Results

**Figure 1:**
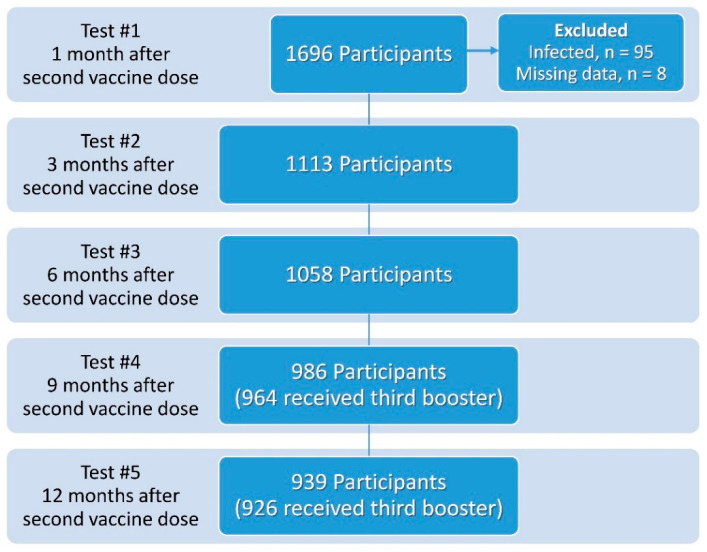
Number of participants at each time point.

In February 2021, one month following the second BNT162b2 vaccine dose, a total of 1696 HCW were evaluated. Of these, 95 were excluded due to SARS-CoV-2 infection any time before the first serology test and 8 were excluded due to missing data. Serology tests were performed at 3, 6, 9, and 12 months after the second vaccine dose in 1113, 1058, 986, and 939 participants, respectively (Figure 1).

The third vaccine dose became available before the fourth test, at month 9. As a result, 964 participants received the third booster dose before undergoing their scheduled (fourth) serology test. Of these, 797 were evaluated at months 9 and 12 for the fourth and fifth serology tests.

Participant characteristics for each of the five serology tests are provided in Table 1.

**Figure 2:**
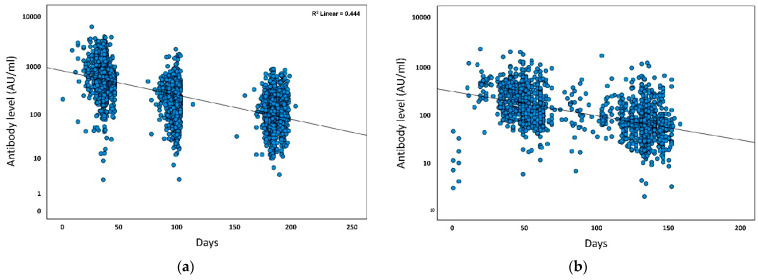
Comparison of antibody concentrations and time for the second and third vaccine doses. (**a**) Time between second vaccine dose and antibody test (days); (**b**) Time between third vaccine dose and antibody test (days).

Antibody concentrations from all five tests are presented in Table 2. Of note, the antibody concentrations (median and means) were higher in tests 4 and 5 as compared to tests 1, 2, and 3. Moreover, there was a greater decrease in antibody concentrations after the second vaccine dose (−8.227, *p* < 0.001, 95% CI −8.798–−7.655 compared to the decrease after the third vaccine dose (−0.852, *p* = 0.034, 95%CI −1.639–−0.065) (Figure 2a,b).

The third vaccine dose was administered at different times for each participant, while the 9 and 12 month tests were pre-scheduled, unrelated to the third dose. Therefore, all tests taken subsequent to receipt of the third vaccine dose were evaluated as continuous variables. A significant inverse correlation was noted between time from third dose and antibody concentrations (Spearman correlation −0.395; *p* < 0.001) (Figure 2b).

The log-transformed antibody concentrations were fit to a mixed effects linear model to define factors that affected the antibody concentrations over time and subsequently adjusted for age, sex, heart disease, malignancy, systemic autoimmune disease, immunodeficiency, hypothyroidism, chronic kidney disease, glucocorticosteroid treatment, and smoking. Age (*p* < 0.0001; CI 95% −0.005–−0.004), heart disease (*p* < 0.0001; CI 95% −0.177–−0.052), immunodeficiency (*p* < 0.0001; CI 95% −0.251–−0.106), and smoking (*p* < 0.0001; CI 95% −0.122–−0.040) were significantly associated with decreased antibody concentrations (Table 3). Female sex (*p* = 0.03; CI 95% 0.013–0.066) was associated with increased antibody concentrations.

## 4. Discussion

This study evaluated the immunogenicity trends among HCW who received a third BNT162b2 vaccination “booster” dose. The waning immunity 6 months after the second vaccine dose is well described [4,5]. Hence, following the spread of the delta variant during July 2021, the Israeli Ministry of Health recommended administering a third booster vaccine dose, first to immunocompromised patients and a month later to patients aged 60 years or older and HCW.

Our findings show that although blood antibody concentrations decreased after the second and third vaccine doses, the decrease was milder, with a smaller slope, after the third vaccine dose than after the second dose. This is encouraging since there is a lack of information regarding the need for several boosters to achieve efficacy for the SARS-CoV-2 infection. The third dose had a substantial effect on morbidity, hospitalization, and mortality [8,9,15]. Our findings are compatible with other reports regarding immunogenicity, and showed a better effect after the third dose than after the first two vaccine doses, mainly in immunocompromised patients [10,11,12]. It merits noting that 3 months after the third booster dose, the antibody concentrations among most of our HCW remained high.

Since emergence of the omicron variant in recent months, knowing that the virus may be contracted despite vaccination has been challenging, although the disease has been milder among vaccinated people [16,17,18,19,20]. Even though the available vaccines may have to be modified to fight new strains of SARS-CoV-2, the fact that the antibodies neutralize the virus is important when considering another vaccine dose.

Our study also found an association between lower antibody concentrations and specific risk factors. Some of these risk factors were described in previous studies, some of which were conducted at our hospital; these risk factors include older age, male sex, and immunodeficiency [21,22,23]. A recent study from Japan found that in addition to immunosuppression, older age, and glucocorticoids, alcohol consumption was also a predictor of lower antibody concentrations among HCW [24]. Age, sex, and immunodeficiency have been described before as risk factors for reduced immune response regarding other vaccines [25]. Our study found two additional risk factors: smoking and heart disease. Smoking along with obesity and hypertension were noted by Watanabe et al. as being associated with lower antibody titers after COVID-19 mRNA vaccines [26]. Another study by Ferrara et al. examined 162 Italian HCW. They also found differences in antibody concentrations between active smokers and non-smokers, as did a systematic review that further supported these findings [27,28]. Smoking and other behaviors such as alcohol consumption have been found to interfere with the response to other vaccines [24,29]. Smoking was related to lower antibody concentrations after vaccination in some studies [30], and was also related to a more severe course of COVID-19 [31]. However, no similar reports could be found regarding our finding that heart disease was inversely related to antibody concentrations. Our finding cannot be explained by information bias, as any misclassification in reporting, if it existed would not be differentiated by antibody concentrations. However, our finding could be related to residual confounding related to other chronic cardiovascular conditions not specified by our study participants as hypertension or other conditions. The relation to heart disease should be further researched.

Our study had some limitations: First, it was a single center study that included HCW who were mostly younger than the general population. Second, neutralizing antibodies were not examined; however, there are studies that have demonstrated the correlation between antibody concentrations and the neutralizing effect [32]. Third, the cellular response was not studied. Nevertheless, we were able to demonstrate with a large cohort a slower decline in immunogenicity after the third booster dose compared to the second dose.

In conclusion, the third booster dose had a better effect on immunogenicity, with higher antibody concentrations among tested HCW. Heart disease and smoking, along with other known risk factors were associated with decreased antibody concentrations. This encouraging data can help in determining vaccine schedules that take into consideration risk factors for lower antibody concentrations, and may help to optimize provision of additional vaccine doses for at-risk populations.

## Figures and Tables

**Table 1:** Participant characteristics for all five serology tests.

Parameter	Test 1 (1 Month) *n* = 1588	Test 2 (3 Months) *n* = 1113	Test 3 (6 Months) *n* = 1058	Test 4 (9 Months) *n* = 964	Test 5 (12 Months) *n* = 926
**Age (mean ± SD)**	47.7 ± 12.6	49.2 ± 12.0	49.9 ± 12.1	50.8 ± 11.7	51.4 ± 11.6
**Sex**					
Male	480 (30%)	297 (27%)	286 (27%)	249 (26%)	252 (27%)
Female	1108 (70%)	816 (73%)	772 (73%)	715 (74%)	674 (73%)
Underlying diseases *					
Heart disease ^†^	56 (3.5%)	39 (3.5%)	42 (4.0%)	41 (4.3%)	35 (3.8%)
Malignancy	22 (1.4%)	17 (1.5%)	16 (1.5%)	17 (1.8%)	16 (1.7%)
Pulmonary disease	57 (3.6%)	42 (3.8%)	45 (4.3%)	37 (3.8%)	37 (4.0%)
Systemic autoimmune disease	109 (6.9%)	95 (8.5%)	83 (7.8%)	83 (8.6%)	78 (8.4%)
Immunodeficiency ^‡^	50 (3.1%)	36 (3.2%)	34 (3.2%)	35 (3.6%)	38 (4.1%)
Hypothyroidism	142 (8.9%)	119 (10.7%)	112 (10.6%)	97 (10.1%)	92 (9.9%)
Chronic renal disease	15 (0.9%)	10 (0.9%)	11 (1.0%)	12 (1.2%)	9 (1.0%)
Dialysis	4 (0.3%)	2 (0.2%)	2 (0.2%)	3 (0.3%)	2 (0.2%)
Other	241 (15.2%)	182 (16.4%)	184 (17.4%)	166 (17.2%)	160 (17.3%)
**BMI ^§^**	*n* = 1082	*n* = 907	*n* = 882	*n* = 888	*n* = 808
<18.5	17 (1.6%)	12 (1.3%)	13 (1.5%)	15 (1.7%)	12 (1.5%)
18.5–24.9	503 (46.5%)	423 (47%)	405 (46%)	404 (45.5%)	364 (45%)
25.0–29.9	363 (33.5%)	309 (34%)	301 (34%)	309 (35%)	285 (35%)
≥30.0	199 (18%)	163 (18%)	163 (18.5%)	160 (18%)	147 (18%)
**Smoker**	119 (7.5%)	94 (8.4%)	99 (9.4%)	89 (9.2%)	75 (8.1%)

* Participants had more than one underlying condition. ^†^ Heart disease included ischemic heart disease and congestive heart disease, but not high blood pressure. ^‡^ Immunosuppressive therapy other than glucocorticosteroids during the last six months. ^§^ Body mass index (BMI) was not provided by all participants.

**Table 2:** Antibody concentrations for each test taken at 1, 3, 6, 9, and 12 months after full (two doses) BNT162b2 vaccination.

Parameter	Test 1 (1 Month) *n* = 1588	Test 2 (3 Months) *n* = 1113	Test 3 (6 Months) *n* = 1058	Test 4 (9 Months) *n* = 964	Test 5 (12 Months) *n* = 926
Mean	683.01	223.75	132.25	2268.15	924.51
Median	568.50	176.00	98.50	1600.00	629.00
Standard Deviation	507.81	197.38	120.17	2301.73	1167.92
Minimum	3	3	4	2	19
Maximum	5920	2020	786	21,400	15,700
Percentiles					
25th	391.50	100.50	53.00	861.50	397.00
50th	568.50	176.00	98.50	1600.00	629.00
75th	792.00	291.50	165.00	2792.50	927.00

**Table 3:** Factors associated with antibody concentrations over time—multivariable analysis from general linear mixed model.

Parameter	Estimate *	*p* Value	95% Confidence Interval	
			Lower Bound	Upper Bound
Age	−0.004	<0.001	−0.005	−0.004
Female sex	0.039	0.003	0.013	0.066
Heart disease ^†^	−0.114	<0.001	−0.177	−0.052
Malignancy	−0.011	0.820	−0.104	0.082
Systemic autoimmune disease	0.010	0.668	−0.037	0.058
Lung disease	0.060	0.055	−0.001	0.121
Immunodeficiency ^‡^	−0.178	<0.001	−0.251	−0.106
Hypothyroidism	0.005	0.824	−0.035	0.044
Chronic kidney disease	−0.053	0.366	−0.168	0.062
Glucocorticosteroid treatment	−0.053	0.366	−0.168	0.062
Smoking	−0.081	<0.001	−0.122	−0.040

Age = random effect. * Estimate represents the statistical importance of fixed-effects coefficient parameters and the slope between the dependent and predictor parameters. ^†^ Heart disease includes ischemic heart disease and congestive heart disease. ^‡^ Immunosuppressive therapy other than gluococorticosteroids during the last six months.

## Data Availability

The data presented in this study are available on request from the corresponding author. The data are not publicly available due to restrictions of privacy of participants.
